# Hemifacial cutaneous sarcoidosis

**DOI:** 10.1016/j.jdcr.2026.05.063

**Published:** 2026-06-04

**Authors:** Abdulelah Aldossari, Mohammed BinMayouf, Ibrahim Alfuraih

**Affiliations:** a Department of Dermatology, King Fahad Specialist Hospital, Buraydah, Saudi Arabia; b Department of Dermatology, King Fahad Medical City, Riyadh, Saudi Arabia

**Keywords:** cutaneous sarcoidosis, facial dermatoses, granuloma, hemifacial, unilateral

## Introduction

Sarcoidosis is a multisystem granulomatous disease of unclear etiology, characterized by noncaseating granulomas and heterogeneous organ involvement.^1^^,^^2^ Diagnosis relies on compatible clinical–radiologic features, histologic confirmation of non-necrotizing granulomatous inflammation, and exclusion of alternative etiologies.^1^

Cutaneous sarcoidosis is a well-recognized manifestation and may be the initial—or occasionally, the only—clinically apparent site of disease.^3^^,^^4^ Its morphologic diversity earns it the designation of “great imitator,” with presentations mimicking inflammatory, infectious, and neoplastic dermatoses.^3^^,^^4^ Skin biopsy provides an accessible route to diagnosis and prompts evaluation for systemic involvement.^4^

Here, we report an unusual presentation of chronic unilateral hemifacial plaque-type sarcoidosis with extension to the ear and chin and with asymptomatic intrathoracic lymph node findings.

## Case presentation

An otherwise healthy 69-year-old Saudi woman, on no prior medications, presented with a 4-year history of slowly progressive, asymptomatic, strictly unilateral facial lesions. She denied constitutional symptoms, respiratory complaints, additional skin lesions, or antecedent facial trauma.

**Fig 1:**
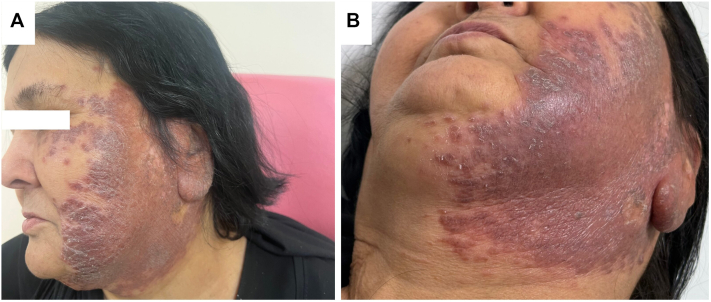
Unilateral violaceous, scaly plaques involving the left face in a segmental distribution. **A,** Close-up view demonstrating contiguous extension to the ipsilateral ear and along the mandibular margin to the chin and submandibular region **(B)**.

**Fig 2:**
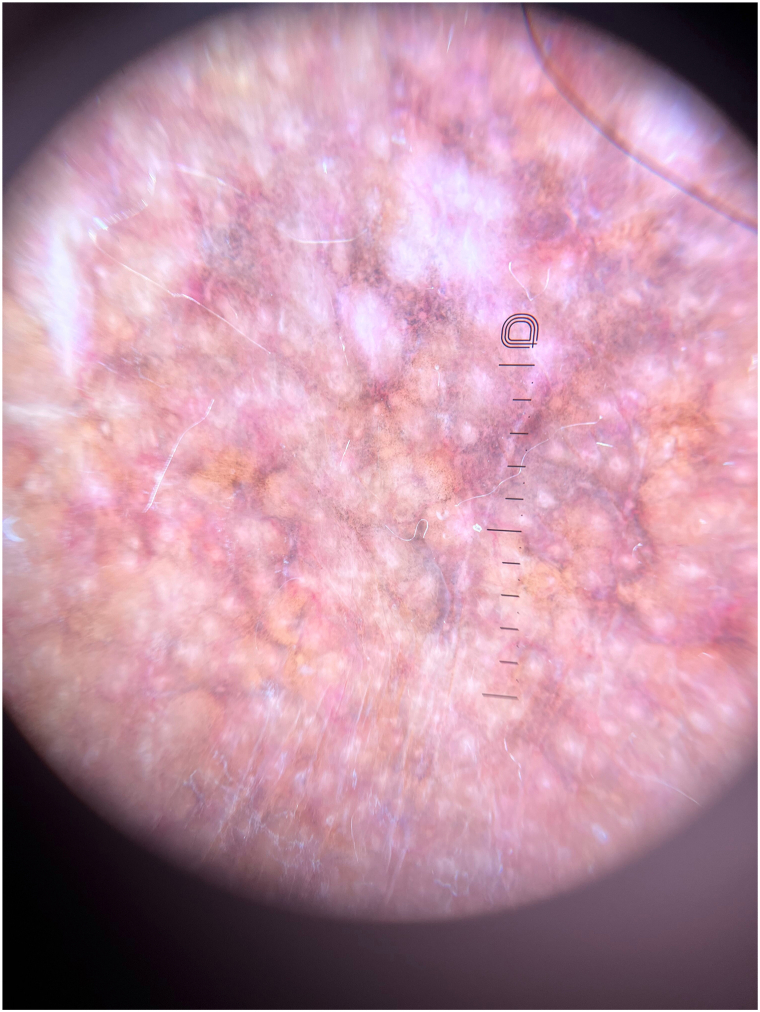
Dermoscopy examination revealed a predominantly structureless *yellow*–*orange* background distributed in multiple ill-defined areas over an erythematous milieu, with scattered whitish structureless areas and subtle fine superficial vessels.

Cutaneous examination demonstrated unilateral, violaceous-to-erythematous indurated plaques with subtle surface scale involving the left face, extending across the preauricular region to the ear and along the mandibular margin to the chin and submandibular area, creating a clinically segmental pattern (Fig 1, *A* and *B*). There was no ulceration, sensory loss, or cranial nerve deficit. Dermoscopy revealed diffuse yellow–orange structureless clods on a pink background (Fig 2). The differential included granulomatous (lupus vulgaris, cutaneous sarcoidosis), vascular, lymphoproliferative, and histiocytic entities.

**Fig 3:**
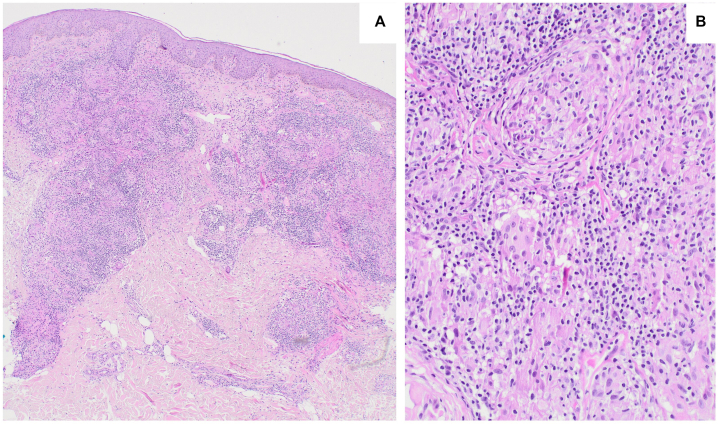
**A** and **B,** Well-formed, non-necrotizing dermal granulomas composed of epithelioid histiocytes and multinucleated giant cells with a surrounding lymphocytic infiltrate; no necrosis, vasculitis, or perineural granulomatous inflammation is present.

A punch biopsy demonstrated dermal nodular aggregates of well-formed non-necrotizing granulomas composed of epithelioid histiocytes and multinucleated giant cells, with a surrounding lymphocytic infiltrate and scattered eosinophils (Fig 3, *A* and *B*). No necrosis, vasculitis, or perineural granulomatous inflammation was identified. Special stains for acid-fast bacilli and fungi (acid-fast bacilli, Periodic acid–Schiff and Grocott-Gomori’s Methenamine Silver) were negative. Tissue cultures were negative for mycobacterial, bacterial, and fungal growth. Overall, the findings supported granulomatous dermatitis, and in the context of chronic unilateral violaceous facial plaques, favored cutaneous sarcoidosis.

Reactive granulomatous dermatitis was considered but excluded by the absence of palisading patterns, drug exposure, or connective-tissue disease on multidisciplinary workup.

Laboratory evaluation revealed an elevated erythrocyte sedimentation rate (38 mm/hr), normal serum calcium and angiotensin-converting enzyme, hepatitis B immunity, and a positive QuantiFERON-TB. High-resolution chest CT demonstrated calcified mediastinal and hilar lymphadenopathy without parenchymal involvement, consistent with radiographic stage I sarcoidosis.^2^ A multidisciplinary team (Pulmonology, Infectious Diseases, Rheumatology) was involved from the outset. Sputum AFB cultures and mycobacterial PCR were negative; the multidisciplinary team attributed the intrathoracic findings to sarcoidosis. Pulmonology elected surveillance and Infectious Diseases initiated latent tuberculosis therapy.

Topical clobetasol propionate 0.05% ointment was initiated; at 1-month follow-up, partial lesion regression was observed. Two sessions of intralesional triamcinolone acetonide (10 mg/cc, then 2.5 mg/cc, 8 weeks apart) were subsequently administered, with topical de-escalation to betamethasone valerate 0.1% ointment twice weekly. No steroid-related adverse effects were observed. Systemic therapy (hydroxychloroquine or methotrexate) was discussed but deferred given the localized, well-responding disease.

**Fig 4:**
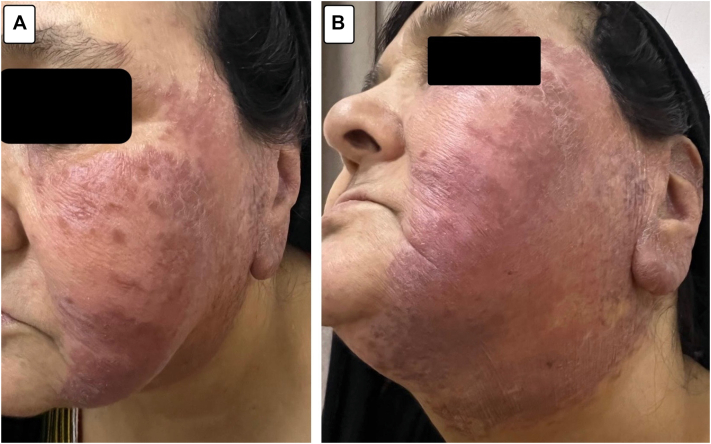
Clinical photographs at the 6-month follow-up visit demonstrating partial response to combined topical and intralesional corticosteroid therapy, with substantial reduction in plaque induration and erythema and residual post-inflammatory dyspigmentation. **A,** Frontal oblique view. **B,** Lateral view of the same patient at the same visit.

At 6-month follow-up, marked reduction in plaque induration and erythema was observed with residual dyspigmentation (Fig 4); no new lesions developed.

## Discussion

Sarcoidosis is a systemic granulomatous disease characterized by noncaseating granulomas affecting multiple organs, most notably the lungs, lymph nodes, and skin, with occasional central nervous system, ocular, or cardiac involvement. Cutaneous disease occurs in approximately 20% of cases and may be the earliest manifestation in up to 88%.^3^^,^^4^ Although cutaneous involvement is frequently accompanied by extracutaneous—particularly pulmonary—disease, increasing evidence supports the view that sarcoidosis may involve a single organ system at any given point in its course, and that isolated cutaneous disease is a legitimate clinical entity.^5^ Diagnosis requires histologic confirmation of non-necrotizing granulomatous inflammation after exhaustive exclusion of infectious, drug-induced, and autoimmune mimics, and cutaneous biopsy remains a highly accessible diagnostic tool.

Cutaneous lesions are classified as specific (sarcoidal granulomas on histology), such as lupus pernio, infiltrative plaques, and scar sarcoidosis, or non-specific (reflecting systemic immunologic phenomena), such as erythema nodosum. Scar sarcoidosis may arise at any site of prior cutaneous trauma or inflammation—including post-herpes zoster sites—making it particularly relevant to the present case.

Liu et al reported facial predilection (71.1%) and plaque predominance (47.4%) in a Taiwanese biopsy-confirmed series.^6^ A strictly unilateral or zosteriform distribution, however, remains distinctly uncommon, reported mainly in isolated cases.^7^ Proposed mechanisms include: (1) Wolf’s isotopic response within a post-herpetic dermatome; (2) Koebnerization at sites of trauma or cosmetic procedures; (3) regional immune dysregulation; and (4) postzygotic cutaneous mosaicism producing a segmental field of heightened granulomatous susceptibility.^8^^,^^9^ In our patient, no antecedent zoster, trauma, or cosmetic procedures were identified and histopathology showed no viral cytopathic features, rendering mosaicism the most attractive—though unconfirmable—hypothesis. VZV PCR was not performed on the biopsy, and a subclinical zoster episode cannot be definitively excluded.^10^

In summary, we present a rare case of hemifacial, quasi-zosteriform plaque-type cutaneous sarcoidosis without a documented antecedent trigger. This case expands the morphologic spectrum of cutaneous sarcoidosis, illustrates the diagnostic utility of dermoscopy and histopathology in segmental facial granulomatous disease, and provides compelling context for the mosaicism hypothesis as a pathogenic alternative to isotopic and Koebnerizing mechanisms. Clinicians should maintain a broad differential for unilateral facial granulomatous plaques and initiate systematic multidisciplinary evaluation.

### Declaration of generative AI and AI-assisted technologies in the writing process

During the preparation of this work the authors used OpenAI to assist with language editing, organization, and grammar accuracy. After using this tool, the authors reviewed and edited the content as needed and take full responsibility for the content of the published article.

## Conflicts of interest

None disclosed.
